# Feasibility and Effectiveness of Using Community Testing Centers to Increase Access to COVID-19 Testing Services in Urban Mozambique

**DOI:** 10.4269/ajtmh.23-0805

**Published:** 2024-11-19

**Authors:** Júlia Sambo, Nádia Sitoe, Neuza Nguenha, Jorfélia Chilaúle, Phath Guambe, Denise Langa, Júlio Rafael, Chishamiso Mudenyanga, Nédio Mabunda, Osvaldo Loquiha, Ilesh Jani

**Affiliations:** ^1^ Instituto Nacional de Saúde, Marracuene, Maputo, Mozambique;; ^2^ Clinton Health Access Initiative, Maputo, Mozambique

## Abstract

Conventional diagnostic systems struggled to meet the fluctuating demand for testing across the different waves of the coronavirus disease 2019 (COVID-19) pandemic. This study aimed to assess the feasibility and effectiveness of the walkthrough (WT) approach in extending access to COVID-19 testing to high-risk populations traditionally underrepresented at health facilities (HFs) and to observe its impact on testing demand.

An interventional study was implemented in markets (WT markets) and ports (WT ports) in Maputo City and Province, Mozambique. Demographic, epidemiological, and clinical data were collected for patients testing for COVID-19 in HFs and at WTs, and a nasal swab COVID-19 antigen rapid diagnostic test (Ag-RDT) was administered.

Overall, testing rates at WTs were higher than those at HFs. At WTs, 4,452 of 4,457 participants were eligible and screened for COVID-19. Most participants were fully vaccinated for COVID-19 and had no symptoms or comorbidities (62.1% at WT markets and 87.9% at WT ports). During the baseline phase, the incidence testing rate of Ag-RDTs in intervention health facilities near the WTs was approximately one-fifth (*P* <0.001) of that in the control HFs located far from the WTs. In the control HFs, the incidence testing rate decreased significantly during the intervention period, whereas in intervention HFs, the incidence testing rate increased by approximately four times (*P* = 0.005).

During times of low positivity rates and limited patient flow, the WT testing points may not yield the expected results in lowering the incidence testing rate within HFs. The WT may constitute an alternative approach to increasing the screening of infectious and noncommunicable diseases.

## INTRODUCTION

Access to timely severe acute respiratory syndrome coronavirus 2 (SARS-CoV-2) testing is crucial to reduce the spread of the virus and the associated morbidity and mortality.^1^^,^^2^ However, in low-income settings, the coverage of laboratory-based diagnostic testing is often limited because of inadequate infrastructure, a lack of equipment, deficient sample referral systems, a shortage of reagents, and a scarcity of trained personnel.^3^^–^^5^ These challenges are further exacerbated during periods of high demand for testing, particularly during epidemics.

During pandemic waves, a long turnaround time (TAT) for results negatively impacts infection control efficiency, especially during periods of high community transmission.^3^^,^^6^ High TATs deter people from seeking diagnostic services and might lead to the continuous transmission of infectious agents in the community. During the coronavirus disease 2019 (COVID-19) pandemic, the utilization of community-based screening points, particularly through the walkthrough (WT) approach, was associated with increased access to testing.^2^^,^^7^^,^^8^

In Mozambique, by the end of 2020, the first year of the response to the COVID-19 pandemic, 68.9% (970,426) of tests were conducted at conventional laboratories using reverse transcriptase polymerase chain reaction (RT-PCR). The use of antigen rapid diagnostic tests (Ag-RDT) was implemented and scaled up to district hospitals to improve testing coverage at high-volume health facilities (HFs) starting in September 2021, during the third COVID-19 wave. In the capital city, Maputo, the WT center approach was implemented at two markets and one bus terminal to reach the population that was unable or unwilling to visit HFs.^2^

We aimed to describe the impact of WT testing centers on the access to diagnosis of high-risk populations in markets and at long-distance truck drivers’ stopping points. We also assessed whether the WT approach had an effect on the number of COVID-19 tests performed in nearby HFs.

## MATERIALS AND METHODS

### Study area and study population.

This study was implemented in high-traffic areas of commuters in Maputo City and Maputo Province, namely, two markets with taxi terminals (Xipamanine and Santos) and two long-distance truck driver stopping points (Maputo Port and Matola Port). The Xipamanine and Matola Santos markets are among the most crowded markets in Maputo City and Maputo Province, respectively. Centro de Saúde de Xipamanine and Centro de Saúde da Matola Santos were the health centers nearest to the selected WT locations. These served as intervention HFs. In selecting control sites, we rigorously evaluated potential HFs based on a set of criteria designed to ensure comparability with intervention sites. These included geographic proximity to high-traffic areas, similar service levels, historical testing volumes, and the socioeconomic and demographic profiles of their respective catchment areas (data not shown). Centro de Saúde de Hulene and Centro de Saúde de Matola 2 were ultimately chosen as control sites because these closely matched the intervention sites across all dimensions, with no WTs nearby (Table 1). The distance from the WT to the control sites was 8.1 km and 5.2 km for the Maputo City and Maputo Province sites, respectively. The distance between the WT and the intervention sites was less than 1 km.

**Table 1:** Walkthrough sites and intervention and control health facilities in Maputo, Mozambique

Facility Type	Type of Site	Site Names
Walkthrough sites	Markets	Xipamanine
		Santos
	Truck driver stopping points	Maputo Port
		Matola Port
Health facilities	Intervention	Centro de Saúde de Xipamanine
		Centro de Saúde de Matola Santos
	Control	Centro de Saúde de Hulene
		Centro de Saúde de Matola 2

Maputo City and Maputo Province are in South Mozambique. In 2022, the estimated populations of Maputo City and Maputo Province were 1,130,319 and 2,390,673, respectively. These two provinces were selected because they reported the highest number of COVID-19-positive cases. The southern area of the country is one of the main corridors for truck drivers because of the Maputo Port and the proximity to South Africa and Eswatini.

COVID-19 testing was made available to all individuals over the age of 18 years who wished to be tested, encompassing both those who met the WHO criteria for COVID-19 testing, including close contacts of confirmed cases, and those without symptoms.^9^ Individuals who had a positive test result for COVID-19 within the previous 7 days were excluded from this study. Testing at WTs was performed between 7:30 am and 3:30 pm from Monday to Friday, excluding public holidays.

This study was implemented from August to November 2022. During this exact period in Mozambique, Vero cell, Pfizer-BioNTech, AstraZeneca, and Janssen from Johnson & Johnson COVID-19 vaccines were authorized and in circulation. The local vaccination guidelines stipulated two doses spaced 4 weeks and 8 weeks apart for the Sinovac and AstraZeneca vaccines, respectively, and a single dose for the Johnson & Johnson vaccine for adults >18 years old.

### Intervention and control HFs.

A primary healthcare facility nearest to the WT testing center was selected as the intervention HF. The control HF was a primary healthcare facility that was similar in terms of the service level. These HFs followed the same routine workflow that was implemented at all HFs offering COVID-19 screening and testing. The HFs tested symptomatic patients and contacts of confirmed cases using rapid diagnostic tests (RDTs).

### Implementation of WT testing centers.

Each WT center had two community mobilizers who raised awareness of the services provided by WTs within the catchment area. People volunteering and consenting to be tested at the WTs had their blood pressure checked, were questioned about any existing chronic conditions, and then offered SARS-CoV-2 Ag-RDT testing using the PanbioTM COVID-19 Ag nasal rapid diagnostic test (Abbott, Jena, Germany, Ref: 41FK11). Each test was preceded by a detailed explanation of the procedure. After receiving their results, individuals were given a comprehensive explanation of what their results meant. Those with positive results had their oxygen saturation checked using handheld pulse oximeters. All patients with positive results were advised to self-isolate for a period of 7 days and encouraged to refer their known close contacts (per the WHO’s definitions) for testing, irrespective of symptoms.^9^ All asymptomatic individuals with negative results were considered negative, whereas all symptomatic individuals with negative results were referred for RT-PCR testing using Abbott m2000 sp/rt platforms (Abbott Molecular Inc., Taipei City, Taiwan), Cobas 6,800 instrument (Roche, Amadora, Portugal), or Applied Biosystem QuantStudio^TM^ 5 Real-Time PCR system (Thermo Fisher Scientific, Waltham, MA) for negativity confirmation (per national guidelines).^10^

Individuals identified as having high blood pressure during the testing process were referred to the nearest HF for a clinical follow-up, regardless of their Ag-RDT results. Those who tested positive for COVID-19 and experienced moderate or severe symptoms, as well as those with low oxygen saturation levels (<95%) and those who reported suffering from hypertension, were referred to COVID-19 treatment centers. Data were collected from the HF near the WT, which served as the intervention arm, and compared with data obtained from the HF in the control arm.

### Data collection, storage, and analysis.

At the WT centers, sociodemographic, clinical, and epidemiological data were collected electronically by using tablets (Samsung, Suwon-si, South Korea, model: TAB A 8”) with Open Data Kit software (v2022.2.1; Seattle, Washington) and stored in a secure database hosted at the Instituto Nacional de Saúde servers. Data were synchronized daily for monitoring by using a dashboard for integrity checking and the resolution of inconsistencies. In the HFs, data were retrospectively collected by using the same instrument as the COVID-19 notification form and patient logbooks.

## STATISTICAL ANALYSES

Descriptive statistics were calculated for the main indicators and summarized in tables and graphs. These indicators included COVID-19 vaccination coverage, which was defined as “fully vaccinated” for the participants who received all the required doses and “incompletely vaccinated” for those who received at least one but not all doses, according to the guidelines. Other indicators were the proportion tested with symptoms, the proportion tested with comorbidities, and Ag-RDT positivity rates. These statistics were displayed for markets, ports, and HFs. Statistical comparisons were made for these indicators between control and intervention HFs to assess WT impact by using a Wald χ^2^ test with robust standard errors.

For the impact of WTs in reducing the testing load in intervention HFs, we compared the Ag-RDT testing rate between control and intervention HFs while controlling for the effects of time (transformed for linearity using a logarithm function), an indicator for baseline data, and a 1-week lagged Ag-RDT positivity rate (i.e., positivity rate from previous week). The Ag-RDT testing rate was defined as the number of COVID-19 tests conducted per 1,000 health facility admissions (any entries at the HFs of the study) per study week, starting from week 1 of August 2022 and continuing until November 2022 (a total of 16 weeks). Using the number of COVID-19 tests per 1,000 HF admissions per week provides a relative measure of testing intensity and the effectiveness of public health interventions. Changes in this rate may signal shifts in testing strategies, changes in the outbreak severity, or changes in the impact of control measures.

As a proxy for the at-risk or exposed population, data from all available admission points at each health facility were used, including emergencies, ambulatory services, and wards. This included all admissions, whether or not they were related to COVID-19, per week, and information was gathered from each service’s logbook. Overall, data were collected from ∼20 admission sites or points in each health facility, with an average of 95 admissions per week. Although HF admission data were used as a proxy for the population at risk, it may not fully capture the entire population at risk of needing COVID-19 testing for various reasons, such as reduced positivity rates leading to negligence about testing, even among those exhibiting symptoms. Thus, this approach likely underestimates the true number of individuals at risk.

We assumed that the number of Ag-RDTs follows from a negative binomial distribution and that the expected counts can be modeled as $\mu_{t}=\exp\;\left(x_{t}\beta +{\mathrm{offset}}_{t}+\upsilon_{t}\right)$, where $\upsilon_{t}$ is the extra Poisson variability, and $e^{\upsilon_{t}}∼\text{Gamma}\;\left(1/\alpha ,\alpha \right)$. The β are regression coefficients for the controlling effects of $x_{t}$, and we used them as the offset or exposure for the total number of patients admitted for any consultation. Baseline data included testing volume records from 3 months before the implementation of the WTs (May to July) obtained from the control and intervention sites. Robust standard errors were used because of the clustering nature of the counts, and data analysis was performed by using STATA/IC 12.1 (StataCorp LLC, College Station, TX) and *R* (R Foundation for Statistical Computing, Vienna, Austria). The alpha (α) value considered was 0.05, corresponding to a 95% confidence level.

## RESULTS

### Characteristics of the study participants.

**Figure 1.**
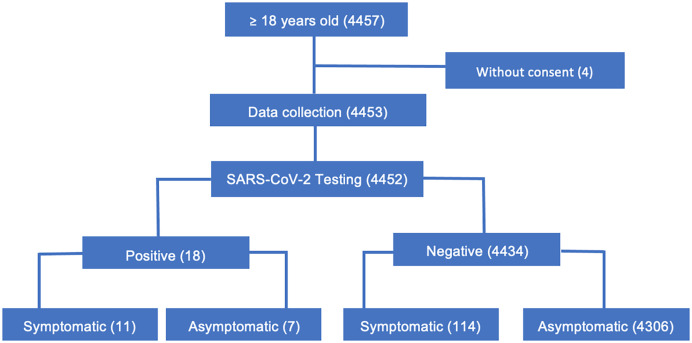
Diagram of participants who attended the walkthrough for coronavirus disease 2019 screening using antigen rapid diagnostic tests.

The four WT enrolled 4,457 participants, of whom 4,452 were eligible and screened for COVID-19 (Figure 1). The median age was 32 years (interquartile range [IQR]: 23–42 years) for participants in WT markets, 39 years (IQR: 34–45 years) for participants in WT ports, 33 years (IQR: 24–46 years) for participants in all HF, 33 years (IQR: 26–46) for participants in intervention HF, and 32 years (IQR: 23–45 years) for participants in control HF. The WT markets and all HFs had more female participants (56.3% and 57.3%, respectively), whereas the WT ports had more male participants (99.1%; Table 2).

**Table 2:** Demographic characteristics of study participants

Characteristic	Markets,* n* = 2,670, 55.5%		Ports,* n* = 1,783, 37.0%		HF,* n* = 361, 7.5%	
	*n*	%	*n*	%	*n*	%
Age groups						
18–29 years	1,202	45.0%	167	9.4%	144	39.9%
30–39 years	514	19.3%	764	42.8%	95	26.3%
40–49 years	368	13.8%	605	33.9%	46	12.7%
50–59 years	274	10.3%	188	10.5%	40	11.1%
60+ years	312	11.7%	59	3.3%	36	10.0%
Median age (IQR)	32 (23–47)		39 (34–45)		33 (24–46)	
Sex						
Female	1,502	56.3%	16	0.9%	207	57.3%
Male	1,168	43.7%	1,767	99.1%	154	42.7%
Nationality						
Mozambican	2,657	99.5%	960	53.8%	343	95.0%
Other	13	0.5%	822	46.1%	0	0.0%
Missing	0	0.0%	1	0.1%	18	5.0%
Residence						
Maputo City	725	27.2%	160	9.0%	72	19.9%
Maputo Province	1,933	72.4%	730	40.9%	252	69.8%
Other province	5	0.2%	40	2.2%	0	0.0%
Missing	7	0.3%	853	47.8%	37	10.2%

HF = health facility; IQR = interquartile range; WT = walkthrough sites.

Participants who attended WT markets (Table 3) were merchants (33.1%), unemployed citizens who lived close to the WT (20.3%), and students (14.6%). Although the participants were mostly vaccinated for COVID-19 (66.9%), a lower proportion (6%) did not complete the vaccination schedule. Most of the tested participants had no symptoms (95.8%), and the overall positivity rate was 0.3% (9/2670; Table 3).

**Table 3:** Description of the participants that attended the WT

Characteristic	Markets, n = 2,670		Ports, n = 1,783	
	*n*	%	*n*	%
Occupation				
Driver/truck driver	61	2.3%	1,691	94.8%
Merchant	885	33.1%	3	0.2%
Unemployed	542	20.3%	3	0.2%
Student	389	14.6%	1	0.1%
Security/army	126	4.7%	40	2.2%
Other	561	21.0%	45	2.5%
Missing	106	4.0%	0	0.0%
COVID-19 vaccination status				
No vaccine	696	26.1%	425	23.8%
Complete vaccination	1,787	66.9%	1,318	73.9%
Incomplete vaccination	161	6.0%	31	1.7%
COVID-19 symptoms				
No	2,557	95.8%	1,758	98.6%
Yes	111	4.2%	13	0.7%
Missing	2	0.1%	12	0.7%
Comorbidities				
Hypertension	296	11.1%	56	3.1%
Gastritis	125	4.7%	24	1.3%
HIV	114	4.3%	21	1.2%
Asthma	79	3.0%	10	0.6%
Rhinitis	51	1.9%	3	0.2%
Other	232	8.7%	32	1.8%
None	1,657	62.1%	1,568	87.9%
Missing	116	4.3%	69	3.9%
Ag-RDT result				
Negative	2,661	99.7%	1,774	99.5%
Positive	9	0.3%	9	0.5%
Missing	0	0.0%	0	0.0%

Ag-RDT = antigen rapid diagnostic test; COVID-19 = coronavirus disease 2019.

In WT ports, participants were mostly truck drivers (94.8%), with security personnel (2.2%) and people with other occupations constituting minor proportions. As observed in the other WT type, the majority of participants (73.9%) were fully vaccinated, and 1.7% did not complete the vaccination schedule for COVID-19. In addition, most of the participants (98.6%) reported no symptoms, and the overall positivity rate was 0.5% (9/1783), which is lower when compared with the positivity rate observed in HFs (Table 3).

The main comorbidities reported by participants in both WT markets and WT ports were hypertension, gastritis, HIV, asthma, and rhinitis (Table 3).

### Impact of WTs on volume and testing rate in intervention HFs.

We conducted our study during a low-transmission period of COVID-19, and in the first week of data collection, a stockout of COVID-19 RDTs was observed in the HF. The overall COVID-19 Ag-RDT positivity rate at HFs was 9.2 (31/337). Higher positivity rates were observed in both control (9.2%, 17/184) and intervention (9.2%, 14/153) HFs, compared with the overall positivity rate at the WTs (0.4%; 18/4453). At WTs, seven out of 18 participants with positive results were asymptomatic (Figure 1).

**Figure 2.**
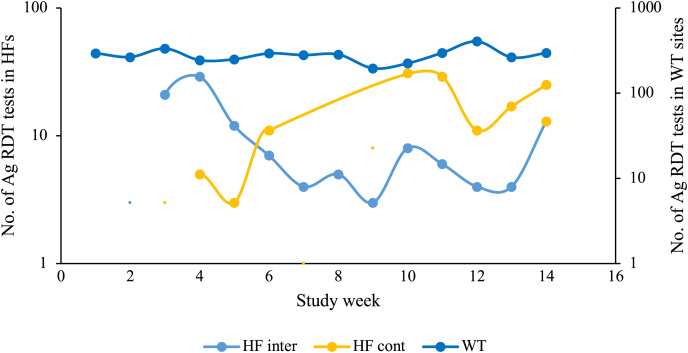
Testing volume measured using the number of antigen rapid diagnostic tests (Ag-RDTs) per study week in control and intervention health facilities (HFs) and walkthroughs (WTs) during the study implementation period. The yellow line represents the HF control, the light blue line represents the HF intervention, and the dark blue line represents the WT sites. The *y*-axis on the left represents the number of Ag-RDTs in HFs, and the *y*-axis on the right represents the number of antigen tests performed in WT sites; both axes are in the logarithm scale (log10). The *x*-axis represents the number of weeks of WT implementation. These are smoothed lines showing the overall tendency of Ag-RDT testing volumes during the study period. cont = control; int = intervention.

Next, we hypothesized that WTs reduced the volume and testing rate of the nearest HFs and measured the testing volumes for COVID-19 in intervention and control HFs. The proportion of participants with symptoms tested in the control HFs (99%; 180/182) was markedly higher than that of intervention HFs (88%; 154/176), and the observed difference was statistically significant (*P* <0.001; Supplementary Table 1). Although testing volumes were constant in the WTs, with a rate of 260 tests per week, we observed different trends in the intervention and control HFs (Figure 2). Starting at week 5, there was an increasing trend of tested individuals in control HFs, with a rate of ∼14 tests per week and a decreasing trend in intervention HFs (rate of 13 tests per week; Figure 2).

**Figure 3.**
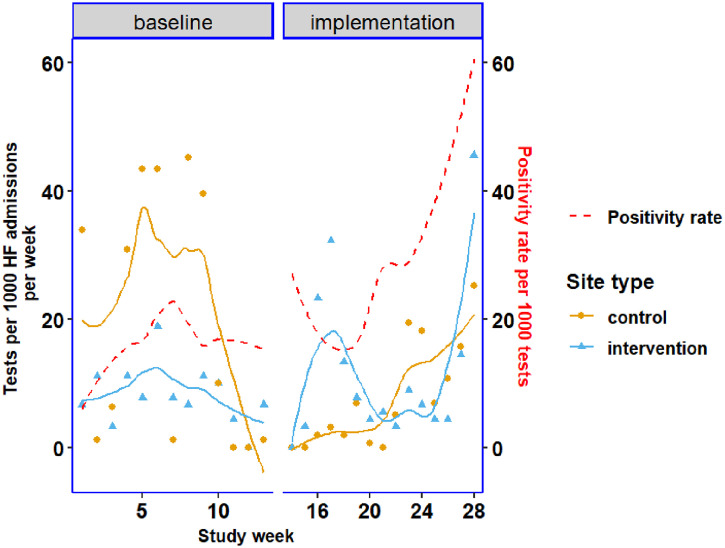
Testing rate by study week for control and intervention sites and by phase (baseline and implementation phase).

We plotted the testing rate, defined as the number of Ag-RDTs per 1,000 HF admissions, by time in weeks (Figure 3). The overall trend during the implementation phase featured an increasing testing rate, showing that in general, the testing rate was relatively similar between the control and intervention sites during this phase (Figure 3), with a higher rate in the intervention sites during the initial weeks of the implementation and a relatively lower rate in the latter weeks. The average testing rate was 8.7 (per 1,000 HF admissions) at control sites and 5.9 (per 1,000 HF admissions) at intervention sites during the implementation period. The positivity rate was relatively lower in the baseline period but increased slightly during the implementation period, with a sharp increase in the latter weeks of the study period, although the positivity rates between the baseline and implementation phases did not differ significantly (Supplemental Table 1 and Figure 3).

The results of the regression model are displayed in Table 4. During the baseline phase, controlling for other factors, such as positivity rate, the incidence rate of Ag-RDTs at intervention sites was roughly 0.2 (95% CI: 0.104–0.361; *P* <0.001) of the incidence rate in the control sites. When comparing the incidence rate of Ag-RDT between the baseline and implementation period at the control sites, the incidence rate during the intervention period was significantly (*P* = 0.019) reduced compared with the baseline period (0.25; 95% CI: 0.082–0.801). Moreover, the incidence rate of Ag-RDT increased by a factor of 3.804 (95% CI: 1.486–9.739) in the intervention HF during the implementation period. The percent change in the incidence rate of Ag-RDTs was a 2.3% increase for every percentage point increase in the Ag-RDT positivity rate (*P* = 0.014). Supplemental Table 2 shows the expected testing rate by site type and phase while keeping the rest of the covariates constant at their mean values.

**Table 4:** Adjusted incidence rate ratio estimates for regression model of Ag-RDT testing rate

Effects	IRR	Robust Std. Err.	95% CI	*P*-Value
Site type				
Baseline period				
Control	ref.			
Intervention	0.194	0.061	0.104–0.361	0.000
Phases				
Control sites				
Baseline	ref.			
Implementation	0.257	0.149	0.082–0.801	0.019
Log time (weeks)	0.857	0.229	0.508–1.445	0.563
Phases, site type				
Implementation, intervention	3.804	1.825	1.486–9.739	0.005
Controlling indicators				
Ag-RDT positivity	1.023	0.009	1.005–1.041	0.014
Constant	0.025	0.011	0.010–0.058	0.000
Alpha	1.099	0.264	0.686–1.758	–
ln (admissions)	1	(exposure)	–	–

Ag-RDT = antigen rapid diagnostic test; IRR = incidence rate ratio.

## DISCUSSION

The present study was implemented in the third year of the COVID-19 pandemic and a year and a half after the initiation of the vaccination process in Mozambique. We hypothesized that community testing centers, named WTs, impact the number of COVID-19 tests performed at the intervention sites compared with the control sites. At the time of the study, numerous preventive and containment measures had already been lifted in the country. Our results show that most of the study population was vaccinated, with a higher percentage observed at WT ports when compared with WT markets. Truck drivers were identified as a high-risk group and potential spreaders of the infection earlier during the pandemic, and special efforts were made to vaccinate them in Mozambique and other African countries.^11^ Furthermore, an important contextual factor for our study was the policy shift in Mozambique in terms of requirements for travelers. Starting in April 2022, before the study period, travelers were no longer required to present a COVID-19 testing certificate but instead had to present a COVID-19 vaccination certificate. Therefore, our study, which was conducted from August to November 2022, provides insights into the impact of point-of-care COVID-19 testing in a period marked by significant policy and behavioral changes related to COVID-19 prevention and control measures. The introduction of vaccination certificates as a requirement for travelers likely influenced public attitudes toward testing and vaccination, potentially affecting the demand for WT testing services. Understanding the timing and implication of this policy change is crucial for interpreting our findings, particularly regarding the observed testing volumes and positivity rates.

The percentage of symptomatic participants and those with positive COVID-19 results was higher in HFs. This can be attributed to the testing algorithm implemented in the country, which stipulated that only patients with symptoms suggestive of SARS-CoV-2 infection or direct contacts of confirmed cases were tested in HFs using Ag-RDTs.^10^ Moreover, a previous evaluation of COVID-19 Ag-RDTs conducted in Mozambique showed that the sensitivity and specificity of these RDTs were higher in symptomatic patients.^11^

Although the WT centers detected 18 cases of COVID-19 out of 4,435 tests, the significance of this intervention extends beyond the detection rate alone. This approach can lead to increased access to testing, the early detection and isolation of positive cases, and increased public health awareness about testing and preventive measures. The WT approach can also contribute to a broader epidemiological understanding of COVID-19’s spread within the community and serve as the cornerstone for community-based public health interventions.

During the implementation of the study, we observed a variation in the COVID-19 positivity rate nationally. The initial weeks of data collection were characterized by a low positivity rate, which gradually increased from midway through the study period. This fluctuation in the positivity rate influenced the testing volume at control HFs, showing an increase that mirrored the country’s positivity rate trend. This national epidemiological context may have influenced the overall incidence rate observed in control HFs during the baseline and implementation periods.

This analysis has shown that during the baseline period, control HFs exhibited a higher incidence rate of COVID-19 testing than implementation HFs. This highlights that although our control and intervention HFs were similar in terms of services delivered, they differed in terms of SARS-CoV-2 diagnosis. This disparity could have been influenced by reported testing disruption due to the stockout of COVID-19 tests observed at both control and implementation HFs, with a more significant impact on the implementation sites. To minimize the impact of the availability of tests, we ensured that all HFs in the study had access to Ag-RDTs for routine testing before the implementation period.

After adjusting the models, we observed an increase in the incidence rate during the implementation period in intervention HFs. These results do not corroborate the positive impact described by other authors in reducing the testing burden on HFs. Our findings indicate that the WT did not reduce the incidence rate in HFs, at least not in the context of low a positivity rate.^2^^,^^8^^,^^12^ Although we initially hypothesized that the WT testing approach could play a significant role in the early detection of COVID-19 cases and potentially reduce community transmission, our findings indicate that the effectiveness of such interventions may be contingent on the prevailing positivity rates within the community. During the study period, observed positivity rates were relatively low, which limited our ability to conclusively demonstrate the anticipated impact of WT testing centers on early case detection and transmission prevention. Therefore, although our results suggest that WT testing centers have the potential to contribute to public health efforts, especially in scenarios of higher transmission, the low positivity rates encountered during the study period offer a cautionary note regarding the conditions under which such interventions are most likely to be effective. It is possible that the true value of WT testing centers in early case detection and the prevention of disease transmission could be more pronounced in contexts with higher baseline levels of COVID-19 or during peaks of transmission, rather than during periods of low positivity, as experienced in our study.^12^^,^^13^ Still, in our study, WTs performed a higher number of tests than HFs, demonstrating not only their feasibility but also increased reach to urban communities. In resource-limited countries, this approach may provide an alternative way to offer healthcare services to communities, such as family planning and screening for noncommunicable diseases.

In comparing ports and markets, a higher number of symptomatic cases was observed in markets. This disparity can be primarily attributed to the differing employment conditions between the two groups commonly frequenting these locations. Specifically, truck drivers who predominantly use ports are often employed by large corporations that offer secure contracts, including benefits such as paid sick leave. This employment stability likely enables truck drivers to adhere more strictly to health guidelines by staying home when symptomatic. Conversely, market sellers and their customers largely consist of self-employed individuals who rely on daily earnings for their livelihood. The absence of a financial safety net compels these informal workers from the markets to continue working, even when they are experiencing symptoms of illness, thereby potentially contributing to the higher observed rate of symptomatic cases in markets. Additionally, individuals exhibiting mild symptoms, or only one symptom, are less likely to seek testing at HFs. However, they still represent a significant group for potential disease transmission.^14^

Although our analysis revealed a 2.3% increase in the incidence testing rate of Ag-RDTs for every percentage point increase in positivity rate, we acknowledge that the overall impact of this finding may be limited given the generally low positivity rate throughout the study period. This incremental change, although statistically significant, should be interpreted within the broader context of testing strategy effectiveness and public health implications.

This study was implemented during a period of decreased positivity rates in the country, immediately following a wave of COVID-19. This fact influenced the number of patients visiting HFs for COVID-19 testing. The testing points (WTs), offering other services, such as blood pressure measurements, may have attracted a larger number of participants. Integrating infectious disease testing with screenings for noncommunicable diseases like hypertension optimizes existing health infrastructure. This dual approach addresses various public health needs simultaneously, enhancing service delivery comprehensively. Such integration broadens the impact of health interventions, meeting a wider range of community health requirements efficiently.

Future studies focusing on the knowledge, attitudes, and practices among the study population, would provide deeper insights into the behavioral factors driving individuals to use these testing facilities, particularly those who are unable or unwilling to visit traditional HFs. Studying these aspects could offer valuable information to refine and enhance public health strategies and interventions, ensuring they are more aligned with the community’s needs and expectations.

## CONCLUSION

During periods of low positivity rates and weak influx, the WT testing points do not have the anticipated effect on reducing the incidence testing rate within HFs. Nevertheless, they might be valuable for pinpointing cases and comorbidity screening in communities. This study demonstrates that public health resources may be better used elsewhere during periods of low COVID-19 transmission in the community.

An appropriate study should be implemented to assess if the WTs can be more effective in situations of high positivity rates, as well as if they can be used to validate vaccination coverage in the general population.

## Supplemental Materials

10.4269/ajtmh.23-0805 Supplemental Materials
