# Supplementary material for: Feasibility and Effectiveness of Using Community Testing Centers to Increase Access to COVID-19 Testing Services in Urban Mozambique
Source: Am J Trop Med Hyg. 2024 Nov 19;112(4 Suppl):46–52. doi: 10.4269/ajtmh.23-0805 (PMC11965724; doi:10.4269/ajtmh.23-0805)
Supplement: Supplemental Materials [file tpmd230805.SD1.pdf]

Supplementary material

Supplementary table 1: Average testing rate (per 1000 hospital admissions), by site type and phase

	Site type			
	Control		Intervention	
Phases	Mean	SE	Mean	SE
Baseline	26.8	7.32	5.8	0.88
Implementation	8.7	2.33	5.9	1.55

SE = standard Error

Supplementary table 2: Predicted testing rate (per 1000 hospital admissions), by site type and phase, adjusted for controlling indicators.

	Site type			
	Control		Intervention	
Phases	Mean	SE	Mean	SE
Baseline	31.3	8.54	6.6	1.05
Implementation	10.5	2.61	10.5	2.61

SE = standard Error
